# Evaluation of Effectiveness of Global COVID-19 Vaccination Campaign

**DOI:** 10.3201/eid2809.212226

**Published:** 2022-09

**Authors:** Daihai He, Sheikh Taslim Ali, Guihong Fan, Daozhou Gao, Haitao Song, Yijun Lou, Shi Zhao, Benjamin J. Cowling, Lewi Stone

**Affiliations:** The Hong Kong Polytechnic University, Hong Kong, China (D. He, Y. Lou);; The University of Hong Kong, Hong Konga (S.T. Ali, B.J. Cowling);; The Laboratory of Data Discovery for Health, Hong Kong Science and Technology Park, Hong Kong (S.T. Ali, B.J. Cowling);; Columbus State University, Columbus, Georgia, USA (G. Fan);; Shanghai Normal University, Shanghai, China (D. Gao);; Shanxi University, Taiyuan, China (H. Song);; Chinese University of Hong Kong, Hong Kong, China (S. Zhao);; RMIT University, Melbourne, Victoria, Australia and Tel Aviv University, Tel Aviv, Israel (L. Stone)

**Keywords:** COVID-19, respiratory infections, severe acute respiratory syndrome coronavirus 2, SARS-CoV-2, SARS, coronavirus disease, zoonoses, viruses, coronavirus, vaccination, vaccine-preventable diseases, global health

## Abstract

To model estimated deaths averted by COVID-19 vaccines, we used state-of-the-art mathematical modeling, likelihood-based inference, and reported COVID-19 death and vaccination data. We estimated that >1.5 million deaths were averted in 12 countries. Our model can help assess effectiveness of the vaccination program, which is crucial for curbing the COVID-19 pandemic.

The real-time evaluation of the effectiveness of vaccination campaigns at the population level is essential for public health policy makers and scientists working toward successful mitigation of the COVID-19 pandemic. Vaccination coverage against SARS-CoV-2 has increased globally and become even more crucial because of the emergence of variants of concern that have increased transmissibility and lethality (*1*). We assessed population-level effects of the COVID-19 vaccination campaign in 12 countries worldwide before November 14, 2021. Our modeling framework enabled us to disentangle the effects of vaccination and a time-varying transmission rate. We also fit the model to multiple waves of death in these countries before the Omicron variant was detected.

## The Study

We developed a transmission modeling approach to analyze diverse spatiotemporal datasets from different countries and attempted to evaluate the COVID-19 vaccination campaign in real time by adapting our related earlier work (*2*). The COVID-19 pandemic continues to be complex because of various short-term enforcements of public health and social measures (e.g., lockdowns), emergence of new virus variants, shifts in age profiles of infected persons, availability of multiple vaccines with different effectiveness, reinfection, and other factors. However, many of these factors are reflected in the key measure, the time-varying transmission rate, *β*(*t*), which characterizes the changes in contact pattern in the population over time. Vaccination is intended to reduce the susceptibility of the population to the disease. Disentangling real-time variation in *β*(*t*) and the effectiveness of vaccination is crucial for assessing the vaccination program and might only be achievable through mathematical modeling.

Country-specific mortality data generally provide a more reliable characterization of the key epidemic dynamics than data on reported confirmed COVID-19 cases, which rely on widely different testing and reporting systems that can vary temporally and spatially and be subject to various ascertainment rates. For our analysis, we obtained data from the World Health Organization, including daily confirmed COVID-19 death numbers (*3*,*4*) and the proportion of the population fully vaccinated (2 doses) for 12 countries: the United Kingdom, Italy, the United States, Spain, Russia, France, India, Brazil, Colombia, Mexico, Germany, and Canada (*5*). We used a partially observed Markov process (*6*) model and maximum-likelihood–based iterative filtering technique to fit and make predictions on the mortality data by susceptible-exposed-infectious-recovered–type models (Appendix). 

We estimated the transmission rate, *β*(*t*), which reflects the simultaneous effect of all possible interventions, excluding vaccination, over the study period. The model assumed a 14-day delay between the 2 vaccine doses and the time for the vaccine to take effect. We set the unified vaccine efficacy (VE; represented by *η*) at 85% and examined vaccine effectiveness from 75% to 95% (Appendix). The COVID-19 surveillance data we used were originally collected from public domains; thus, neither ethical approval nor patient consent was applicable. 

To evaluate effectiveness of vaccination and the lives saved, we compared the final model fit and simulations of the baseline scenario of vaccination to the counterfactual scenario of without vaccination by setting VE to *η* = 0. Vaccination coverage was defined as the proportion of the country’s population that was fully vaccinated (i.e., either receiving 2 vaccine doses or receiving 1 vaccine dose after infection). We plotted vaccination coverage as a function of time for the 12 countries (Appendix Figure 1).

We compared and fitted the model to data on weekly confirmed waves of COVID-19 deaths in the 12 countries during 2020–2021 and reconstructed transmission rates (Appendix Figure 1, panels A–I). We then used the model to reconstruct COVID-19 deaths that would have occurred in these countries in the hypothetical without-vaccination counterfactual scenario (i.e., in complete absence of vaccination). Thus, we could compare the observed mortality rate against that of the model’s without-vaccination scenario (Appendix Figure 1).

We found that vaccination campaigns saved the lives of up to 1,822,670 (0.069% of the total population) persons in these 12 countries (Appendix Table 2). For instance, the United States reported 416,842 confirmed deaths during January 1–November 14, 2021 (Appendix Figure 1, panel E). According to the model’s without-vaccination predictions, had the United States not initiated a vaccination program, 1,102,958 deaths would have occurred there during the same time frame. Thus, vaccination saved 686,115 lives (0.2% of the population) in the United States during the study period. The model estimated that vaccination averted 182,464 (0.27% of the population) deaths in the United Kingdom; 109,367 (0.23% of the population) deaths in Spain; 78,969 (0.2% of the population) deaths in Canada; and 96,008 (0.16% of the population) deaths in Italy. Vaccination coverage in each of these countries was >60% (Appendix Table 2).

Vaccination seems to have prevented severe Delta waves in Italy, France, Germany, and Canada during the second half of 2021 (Appendix Figure 1). For Russia, India, Brazil, Colombia, and Mexico, where vaccine coverage was relatively low or delayed, vaccination had only a mild effect on the epidemic dynamics and mortality rates (Appendix Table 2).

 Widely available vaccines might encourage risky behavioral practices among the population, which might be less prevalent in the absence of a countrywide vaccination campaign. Our idealized reconstruction method ignores this possibility and might have led to overestimation of both the transmission rate in the without-vaccination scenario and the number of deaths averted (*7*). To examine this possibility further, we plotted the changes in deaths averted by vaccination as a percentage of the population as calculated for 5 levels of transmission rate reduction (Figure). The reductions are intended to compensate for risky behaviors persons might engage in when vaccinated. We considered these as 5 counterfactual without-vaccination scenarios in which transmission rates after April 16, 2021, were reduced to 0 (scenario 1), 10% (scenario 2), 15% (scenario 3), 20% (scenario 4), and 50% (scenario 5) of the level of transmissibility in the baseline scenario. These counterfactual scenarios were intended to show that any overestimation of deaths averted based on the idealized counterfactual scenario 1 (0 reduction) was generally minimal unless the transmission rate was reduced by >25% (Appendix).

**Figure:**
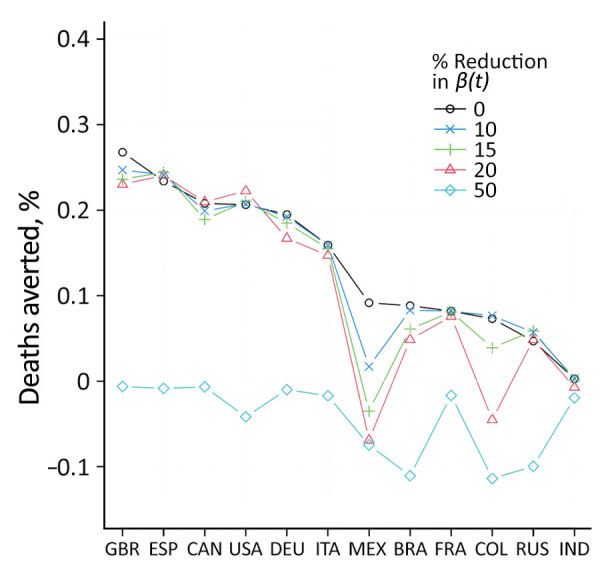
Deaths averted because of vaccination according to a model used to evaluate effectiveness of global COVID-19 vaccination campaign. The graph represents the difference in total deaths under the counterfactual scenario (without vaccination) and under the baseline scenario (with vaccination) as a percentage of the population. We compared 5 counterfactual scenarios under without-vaccination in which we set the transmission rates after April 16, 2021, to reduce by 0, 10%, 15%, 20%, and 50% compared with the baseline scenario. The y-axis 0.3% means 3 persons per 1,000 population were saved from COVID-19–related death because of vaccination. The absolute value of negative deaths averted results from substantial reduction in transmission rate, rather than vaccination. *β*(*t*), time-varying transmission rate; BRA, Brazil; CAN, Canada; COL, Colombia; DEU, Germany; ESP, Spain; FRA, France; GBR, Great Britain (United Kingdom); IND, India; ITA, Italy; MEX, Mexico; RUS, Russia; USA, United States.

We conducted additional sensitivity analyses on the model performance and counterfactual scenarios to explore parameter ranges and several different model structures, constructing more complex models of imperfect vaccination (Appendix Tables 1–3, Figures 2, 3). Our estimates of deaths averted show reasonable robustness to changes in the model structure and parameters.

## Conclusions

We used a disease transmission model and likelihood-based inference approach to evaluate effectiveness of COVID-19 vaccination in 12 countries. Our analysis indicated that vaccination averted >1.5 million deaths in the studied countries until November 14, 2021, or at least precluded the need to reintroduce more stringent public health and social measures to control transmission. 

Of our several assumptions for this evaluation, we first assumed the infection fatality ratio was roughly constant over time (*1*,*8*,*9*). We evaluated a second model in which we allowed the infection fatality ratio to decrease because of vaccination (Appendix). In addition, we used a unified constant VE although VE differs across countries, demographic characteristics (*10*), and type of vaccine and its coverage (*11*). Nonetheless, our modeling framework enabled us to assess the effect of vaccination on a time-varying transmission rate. Our model can help assess effectiveness of the COVID-19 vaccination program, which is crucial for curbing the COVID-19 pandemic. 

Appendix Additional information on evaluation of the effectiveness of global COVID-19 vaccination campaign.
