# Supplementary material for: Evaluation of Effectiveness of Global COVID-19 Vaccination Campaign
Source: Emerg Infect Dis. 2022 Sep;28(9):1873–6. doi: 10.3201/eid2809.212226 (PMC9423931; doi:10.3201/eid2809.212226)
Supplement: Appendix — Additional information on evaluation of the effectiveness of global COVID-19 vaccination campaign. [file 21-2226-Techapp-s1.pdf]

Article DOI: https://doi.org/10.3201/eid2809.212226

Evaluation of Effectiveness of Global COVID-19 Vaccination Campaign

Appendix

Additional Methods

Transmission Models and Fitting

We make use of the classic susceptible-exposed-infectious-recovered (SEIR) model. The rate at which the susceptible population is vaccinated is denoted by $\tilde{v}(t)$. Studies note that vaccination induces different levels of protection, which are associated with different risks of breakthrough infection. For simplicity, we assume that vaccinated susceptible persons can be divided into 2 groups: a high protection group and a low protection group. The high protection group is assumed to enter the recovered class (R) and gain long-term full protection after vaccination. There are different approaches to deal with the low protection group. Thus, we analyzed 2 different models based on the 2 different ways to deal with the low protection group. In model 1, the low protection group comprises persons who remain in the Susceptible (*S*) class after vaccination and are available for infection and possibly revaccination; in model 2, the low protection group comprises persons who enter the V class after vaccination, where they become available for infection at reduced susceptibility.

Model 1

The equations of model 1 are derived from previous studies (*1–3*). Model 1 is:

$$\dot{S} = -\frac{\beta SI}{N} - \eta\tilde{v}S,$$
$$\dot{E} = \frac{\beta SI}{N} - \sigma E,$$
$$\dot{I} = \sigma E - \gamma I,$$
$$\dot{H} = \pi\gamma I - \kappa H,$$
$$\dot{D} = \theta\kappa H,$$
$$\dot{R} = \eta\tilde{v}S + (1-\pi)\gamma I + (1-\theta)\kappa H.$$

where *S*, *E*, *I*, *H*, *D*, and *R* denote the mean number of the population that are susceptible (*S*), exposed (*E*), infectious (*I*), a delayed class of hospitalized (*H*) persons between infectious (*I*) and dead (*D*) and recovered (*R*), respectively. η is the proportion that become fully protected after vaccination (a proxy measure of vaccine efficacy). β is the transmission rate, σ is the rate at which exposed persons become infectious, γ is the recovery rate, π is the proportion of infectious persons that enter the delayed class *H*, κ the rate persons discharged from the delayed *H* class, and θ the proportion of deaths of the discharged persons. We suppose that reinfection and breakthrough infections are not significant in terms of contributing to deaths, which is a reasonable first approximation.

Following the literature, $\sigma = 0.5$ per day, $\gamma = 0.33$ per day and $\kappa = 0.0833$ per day, such that the mean generation time (sum of mean latent period and mean infectious period) is 5 days (*4*,*5*), and the mean duration from infection to death is 17 days, which are largely in line with observations (*6*). It is assumed that $\pi = 0.15$ for countries other than India where $\pi = 0.03$ (*7*,*8*), and β and θ are estimated through fitting. The choice of π is not our immediate interest because we do not fit data for *H*; hence it is not dealt with in our fitting exercise. The infection fatality rate (IFR) = $\theta\pi$. The choice of $\pi = 0.03$ in India was based on considerations of the infection to case ratio there (as high as 24:1), low reported deaths and high estimates of seroprevalence in India (*8*). Namely the reported deaths are relatively low per capita and all serologic studies in India suggest a large proportion of the population have been infected. We assume θ is in the range of 0.02–0.04, which means an IFR in the range of 0.3%–0.6% in the 11 countries except for India, which is reasonable (*7*). The IFR in India is 1/5 of that in the other 11 countries (*8*).

We allowed the time-dependent transmission rate, $\beta(t)$, to be estimated by an exponential cubic spline (*9*) with several nodes $n_\beta = 10$ and an upper limit of 608, such that the reproduction number (without vaccine) is in a reasonable range with an upper limit of 5. The choice of cubic

spline was the same as in our previous studies in modeling multiple waves of infections (*10–12*). Alternatively, one could use the mobility index data in the transmission instead of cubic spline. However, the mobility index data alone are insufficient (*7*,*13*,*14*). The emergence of new variants with increased transmissibility will increase the overall transmissibility in our 1-strain model.

Because the risk for infection is not uniform in the population and some persons might have strong protection compared with others, we assumed for initial conditions that 5% of the population were somehow protected or possibly had pre-existing cross-immunity from other coronaviruses (*15*). The initial E and I populations were equal and randomly chosen in the range of 0–10,000. The H class was given 1/10 the population of the I class, and the D class had 1/10 the population in the H class.

A partially observed Markov process (POMP) model with a maximum likelihood based iterated filtering technique was used to fit the mortality data (*11*). As mentioned, the transmission rate, $\beta(t)$, was taken as an exponential cubic spline (*9*) to account for the simultaneous impact of all possible interventions excluding vaccination. The fitting procedure can be found at https://kingaa.github.io/sbied. Appendix Figure 1 shows the fitting and simulation results of model 1 with vaccine efficacy (VE) set at 85%.

Model 2

In model 2, we extended model 1 by including an additional vaccinated compartment (V) for tracking the dynamics of vaccinated but only partially susceptible persons (*16*). Thus, we further consider reduced susceptibility, reduced fatality rate due to vaccination, or both. The equations for model 2 are:

$$\dot{S} = -\frac{\beta SI}{N} - \tilde{v}S,$$

$$\dot{V} = (1-\eta)\tilde{v}S - \frac{\psi\beta VI}{N},$$

$$\dot{E} = \frac{\beta SI}{N} + \frac{\psi\beta VI}{N} - \sigma E,$$

$$\dot{I} = \sigma E - \gamma I,$$

$$\dot{H} = \pi\gamma I - \kappa H,$$

$\dot{D} = \theta\kappa H,$

$\dot{R} = \eta\tilde{v}S + (1-\pi)\gamma I + (1-\theta)\kappa H,$

Here, ψ is the parameter that accounts for the reduced susceptibility of vaccinated persons, where $0<\psi\leq 1$. We show fitting results of model 2 with $\psi = 0.6$ (Appendix Figure 2).

Vaccination Rate

We downloaded data for the vaccination rate, $v(t)$, from the Our World in Data Web site (*17*,*18*), which is the proportion of the whole population vaccinated per unit of time. First, we calculated $\tilde{v}(t)$, the proportion of susceptible persons vaccinated per unit of time. The population is divided into 2 groups, vaccinated and unvaccinated; vaccination is only delivered to the unvaccinated group, which includes both susceptible and recovered persons. The rate at which susceptible persons are vaccinated is given as

$$\tilde{v}(t) = v(t)/(1 - \int_0^{t-1} v(s)\mathrm{ds})$$

where t is in units of days (*1–3*,*16*). We assume a delay of 14 days between the delivery of the second vaccine dose and the onset of protection, thus:

$$\tilde{v}(t+14) = v(t)/(1 - \int_0^{t-1} v(s)\mathrm{ds}).$$

Asymptomatic Cases

A large proportion of infections are asymptomatic and less infectious than symptomatic cases, as reported in our earlier works (*19*,*20*). However, we adopted a simple homogeneous model that aggregates both the symptomatic and asymptomatic cases following other previous studies, such as Yang and Shaman (*13*).

Discussion

It is possible that population behavioral patterns become more careless and unstable due the widespread availability of vaccines over time (*21*). This might modulate the transmissibility across the epidemic and consequently cause us to overestimate the total deaths averted because of a vaccination campaign. To assess the effects of the vaccination, we compared the scenarios of with-vaccination (baseline scenario) and without-vaccination (counterfactual scenario). To test the sensitivity of varied transmissibility, we considered 5 sets of simulations all without-

vaccination ($v(t) = 0$), but the transmission rate after April 16, 2021 was reduced by 0%, 10%, 15%, 20%, and 50% of the baseline scenario's level (reconstructed transmissibility from data with vaccination). We plotted the number of deaths that would have been averted as a percentage of the total population of each country with model 1 (VE = 85%) and these 5 counterfactual scenarios on transmissibility (Figure in main text).

Thus, if the reduction in model transmissibility is very large, say 50%, the disease will go extinct and few persons will die, which is not so different to the scenario under a successful vaccination policy. As such, we saw virtually no difference between the model simulations with 50% transmission reduction and what happened in all vaccinated countries (Figure in main text), because the vaccinations averted many of the possible deaths. Thus, the difference in deaths averted for the 2 scenarios appears as ≈0%.

If no reduction in transmission occurs (i.e., 0% reduction graph in Figure in main text) but vaccination is switched off, then most countries have major epidemics in this scenario and the differences in deaths averted is major compared to the vaccinated baseline. This is the scenario we discuss in the main text and which we are testing for possible overestimation.

We examined what happens in between those 2 extremes. Of note, according to Figure in main text, a 20% transmission reduction is not enough to bring about disease extinction and the I class is still able to grow exponentially in some phases for many countries. As such, we saw that for many countries, such as the United Kingdom, Spain, Germany, the United States, Italy, and France, this 20% reduction in transmission is not very different in terms of deaths averted than the 0% transmission reduction, and implies our overestimation not too large. We saw <15% difference in the deaths averted in 8 of the 12 countries, namely the United Kingdom, Italy, Russia, France, the United States, Spain, Germany, and Canada. On the other hand, with the 20% transmission reduction in 2 countries, Mexico and Columbia, the herd immunity threshold was crossed and the disease rapidly became extinct. This indicates that a 20% reduction in the transmission rate is probably too large to be reasonable, and that level of reckless behavior is unrealistic, which was confirmed by examining a scenario of 25% transmission reduction, which led to disease extinction in most countries.

Other than the above, we know of no other method to explore the effects of reckless behavior that might lead to overestimations but recognize this as a possible limitation of the method.

Sensitivity Analysis

In the above, we consider model 1 with VE = 85% and model 2 with susceptibility reduction $\psi = 0.6$. Here, we consider variations in the model. We consider model 1 with VE = 75% and VE = 95%. We consider model 2 with $\psi = 0.8$. In model 2, we further replace θ with the following equation:

$$\tilde{\theta} = \left(1 - \varepsilon \int_0^t v(s)\, ds\right) \theta$$

Namely, we assume that the death rate, $\tilde{\theta}$, drops while the proportion of vaccinated persons increases at a rate of the following:

$\int_0^t v(s)\, ds,$ in which limit $\int_0^T v(s)\, ds = 1$

and the death rate, $\tilde{\theta}$, could drop by $\varepsilon = 25\%$. All together, we have 6 model variations (Appendix Table 1).

We fit these 6 model variations (including our baseline model, which is model 1 version 1 with VE = 85%) to the respective data to find the maximum-likelihood parameter estimates. All model variations fit the data reasonably well (Appendix Figure 2). We compared the model-estimated deaths in 2021 (up to November 14, 2021) in 12 countries under the 6 model variations (Appendix Tables 2, 3; Appendix Figure 3), together with the first counterfactual scenario of without-vaccination, $v(t) = 0$.

We found that the 12 countries fall in 2 groups, the first group of counties (including the United Kingdom, Spain, Canada, the United States, Germany, and Italy), had 0.1%–0.3% of their population saved from death while the second group of countries (including Mexico, Brazil, France, Colombia, Russia, and India) had <0.1% of their population saved. This pattern is insensitive to parameter values we considered, despite substantial changes across 6 model variations.

From this and a closer examination (Appendix Tables 2, 3), we concluded that our estimates of deaths averted show reasonable robustness to changes in the model structure and

parameters. We have further confirmed this with a study of much larger number of model variations than reported here.

Reference

1. Song H, Fan G, Liu Y, Wang X, He D. The second wave of COVID-19 in South and Southeast Asia and the effects of vaccination. Front Med (Lausanne). 2021;8:773110. PubMed https://doi.org/10.3389/fmed.2021.773110

2. Musa SS, Tariq A, Yuan L, Haozhen W, He D. Infection fatality rate and infection attack rate of COVID-19 in South American countries. Infect Dis Poverty. 2022;11:40. PubMed https://doi.org/10.1186/s40249-022-00961-5

3. Lin L, Chen B, Zhao Y, Wang W, He D. Two waves of COIVD-19 in Brazilian cities and vaccination impact. Math Biosci Eng. 2022;19:4657–71. PubMed https://doi.org/10.3934/mbe.2022216

4. Tang X, Musa SS, Zhao S, Mei S, He D. Using proper mean generation intervals in modeling of COVID-19. Front Public Health. 2021;9:691262. PubMed https://doi.org/10.3389/fpubh.2021.691262

5. Griffin J, Casey M, Collins Á, Hunt K, McEvoy D, Byrne A, et al. Rapid review of available evidence on the serial interval and generation time of COVID-19. BMJ Open. 2020;10:e040263. PubMed https://doi.org/10.1136/bmjopen-2020-040263

6. de Souza WM, Buss LF, Candido DDS, Carrera J-P, Li S, Zarebski AE, et al. Epidemiological and clinical characteristics of the COVID-19 epidemic in Brazil. Nat Hum Behav. 2020;4:856–65. PubMed https://doi.org/10.1038/s41562-020-0928-4

7. Unwin HJT, Mishra S, Bradley VC, Gandy A, Mellan TA, Coupland H, et al. State-level tracking of COVID-19 in the United States. Nat Commun. 2020;11:6189. PubMed https://doi.org/10.1038/s41467-020-19652-6

8. Murhekar MV, Bhatnagar T, Thangaraj JWV, Saravanakumar V, Kumar MS, Selvaraju S, et al.; ICMR Serosurveillance Group. SARS-CoV-2 seroprevalence among the general population and healthcare workers in India, December 2020-January 2021. Int J Infect Dis. 2021;108:145–55. PubMed https://doi.org/10.1016/j.ijid.2021.05.040

9. Press WH, Teukolsky SA, Vetterling WT, Flannery BP. Numerical recipes with source code CD-ROM 3rd edition: the art of scientific computing. Cambridge: Cambridge University Press; 2007.

10. Bretó C, He D, Ionides EL, King AA. Time series analysis via mechanistic models. Ann Appl Stat. 2009;3:319–48. https://doi.org/10.1214/08-AOAS201

11. Ionides EL, Bretó C, King AA. Inference for nonlinear dynamical systems. Proc Natl Acad Sci U S A. 2006;103:18438–43. PubMed https://doi.org/10.1073/pnas.0603181103

12. Lin Q, Chiu AP, Zhao S, He D. Modeling the spread of Middle East respiratory syndrome coronavirus in Saudi Arabia. Stat Methods Med Res. 2018;27:1968–78. PubMed https://doi.org/10.1177/0962280217746442

13. Yang W, Shaman J. Development of a model-inference system for estimating epidemiological characteristics of SARS-CoV-2 variants of concern. Nat Commun. 2021;12:1–9. https://doi.org/10.1038/s41467-021-27703-9

14. Yang W, Shaman J. COVID-19 pandemic dynamics in India, the SARS-CoV-2 Delta variant, and implications for vaccination. J R Soc Interface. 2022;19:20210900. PubMed https://doi.org/10.1098/rsif.2021.0900

15. Ng KW, Faulkner N, Cornish GH, Rosa A, Harvey R, Hussain S, et al. Preexisting and de novo humoral immunity to SARS-CoV-2 in humans. Science. 2020;370:1339–43. PubMed https://doi.org/10.1126/science.abe1107

16. Lin L, Zhao Y, Chen B, He D. Multiple COVID-19 Waves and Vaccination Effectiveness in the United States. Int J Environ Res Public Health. 2022;19:2282. PubMed https://doi.org/10.3390/ijerph19042282

17. Mathieu E, Ritchie H, Ortiz-Ospina E, Roser M, Hasell J, Appel C, et al. A global database of COVID-19 vaccinations. Nat Hum Behav. 2021;5:947–53. PubMed https://doi.org/10.1038/s41562-021-01122-8

18. Ritchie H, Mathieu E, Rodés-Guirao L, Appel C, Giattino C, Ortiz-Ospina E, et al. Coronavirus pandemic (COVID-19). Our World in Data. [cited 2022 May 1]. https://ourworldindata.org/coronavirus.

19. Wu P, Liu F, Chang Z, Lin Y, Ren M, Zheng C, et al. Assessing asymptomatic, presymptomatic, and symptomatic transmission risk of severe acute respiratory syndrome coronavirus 2. Clin Infect Dis. 2021;73:e1314–20. PubMed https://doi.org/10.1093/cid/ciab271

20. He D, Zhao S, Lin Q, Zhuang Z, Cao P, Wang MH, et al. The relative transmissibility of asymptomatic COVID-19 infections among close contacts. Int J Infect Dis. 2020;94:145–7. PubMed https://doi.org/10.1016/j.ijid.2020.04.034

21. Newman PA, Lee SJ, Duan N, Rudy E, Nakazono TK, Boscardin J, et al. Preventive HIV vaccine acceptability and behavioral risk compensation among a random sample of high-risk adults in Los Angeles (LA VOICES). Health Serv Res. 2009;44:2167–79. PubMed https://doi.org/10.1111/j.1475-6773.2009.01039.x

Appendix Table 1. Variations in parameter settings for models used to evaluate of effectiveness of global COVID-19 vaccination campaign*

Model	Variation	η	ψ	ε
Model 1	1	0.85	–	–
	2	0.75	–	–
	3	0.95	–	–
Model 2	1	0.85	0.6	0
	2	0.85	0.8	0
	3	0.85	0.8	0.25

*Model 1 is a Susceptible-Exposed-Infectious-Hospitalized-Died-Recovered (SEIHDR) model; model 2 is an extension of model 1 in which the vaccinated group (V) has reduced susceptibility (controlled by ψ) and reduced death rates (controlled by ε). η is the proportion of the population that becomes fully protected after vaccination, a proxy measure of vaccine efficacy.

Appendix Table 2. Estimated effects of vaccination on COVID-19 mortality in 12 countries during period January 1–November 14, 2021, according to model 1 used to evaluate of effectiveness of global COVID-19 vaccination campaign*

	Version 1			Version 2			Version 3		
	Estimated deaths		Lives saved, %†	Estimated deaths		Lives saved, %†	Estimated deaths		Lives saved, %†
Countries	With vaccination	Without vaccination		With vaccination	Without vaccination		With vaccination	Without vaccination	
United Kingdom	60,866	243,330	0.268	60,486	232,982	0.253	59,432	249,693	0.279
Spain	32,937	142,304	0.234	32,944	118,640	0.183	32,712	166,732	0.287
Canada	13,561	92,530	0.208	13,122	91,132	0.205	11,929	105,212	0.246
United States	416,842	1,102,958	0.206	415,762	902,871	0.147	418,884	828,990	0.123
Germany	65,806	229,486	0.195	65,742	241,046	0.209	65,176	198,762	0.159
Italy	59,262	155,270	0.159	59,400	136,902	0.128	58,641	162,886	0.173
Mexico	170,352	289,428	0.092	136,645	234,134	0.075	129,716	240,521	0.085
Brazil	404,648	593,256	0.088	408,048	552,938	0.068	407,292	582,875	0.082
France	46,446	100,016	0.082	46,829	93,682	0.072	46,746	104,241	0.088
Colombia	85,132	122,628	0.073	86,039	115,024	0.057	85,350	127,294	0.082
Russia	201,322	269,720	0.047	199,641	261,862	0.043	203,734	281,979	0.054
India	297,380	336,300	0.003	299,676	329,029	0.002	301,682	339,829	0.003

*Model-simulated COVID-19 deaths under the the actual, with vaccination, and the first counterfactual scenarios with different parameter choices. In version 1, η = 0.85; in version 2, η = 0.75; and in version 3, η = 0.95, in which η is the proportion of the population that becomes fully protected after vaccination, a proxy measure of vaccine efficacy.
†Deaths averted as a percentage of country's population.

Appendix Table 3. Estimated effects of vaccination on COVID-19 mortality in 12 countries during period January 1–November 14, 2021, according to model 2 used to evaluate of effectiveness of global COVID-19 vaccination campaign*

	Version 1			Version 2			Version 3		
	Estimated deaths		Lives saved, %†	Estimated deaths		Lives saved, %†	Estimated deaths		Lives saved, %†
Country	With vaccination	Without vaccination		With vaccination	Without vaccination		With vaccination	Without vaccination	
United Kingdom	60,590	244,324	0.27	59,600	238,503	0.262	60,782	238,922	0.261
Spain	32,969	136,104	0.221	33,112	115,904	0.177	32,836	126,252	0.2
Canada	9,796	126,618	0.307	12,683	108,428	0.252	10,314	160,966	0.397
United States	421,184	784,416	0.109	418,939	982,228	0.169	411,548	1,177,809	0.23
Germany	67,052	282,824	0.257	65,364	238,346	0.206	66,072	291,765	0.269
Italy	59,604	139,901	0.133	59,532	177,586	0.195	59,624	151,994	0.153
Mexico	133,740	230,597	0.075	153,000	260,745	0.083	142,408	244,554	0.079
Brazil	408,042	522,342	0.053	407,606	583,784	0.082	407,471	573,858	0.078
France	47,396	111,377	0.098	46,915	102,264	0.085	47,588	96,064	0.074
Colombia	85,752	123,214	0.073	85,079	119,061	0.066	84,812	119,596	0.068
Russia	201,765	271,726	0.048	202,200	275,159	0.05	204,876	279,038	0.051
India	322,055	356,146	0.002	292,800	327,646	0.003	296,065	336,642	0.003

*Model-simulated COVID-19 deaths under the actual, with vaccination, and the first counterfactual scenarios with different parameter choices. In version 1, ψ = 0.6; in version 2, ψ = 0.8; in version 3, ψ = 0.8, and $\tilde{\theta}$ replaces θ. ψ, model parameter that accounts for the reduced susceptibility of vaccinated persons; θ, proportion of deaths of persons discharged from hospitals.
†Deaths averted as a percentage of country's population.

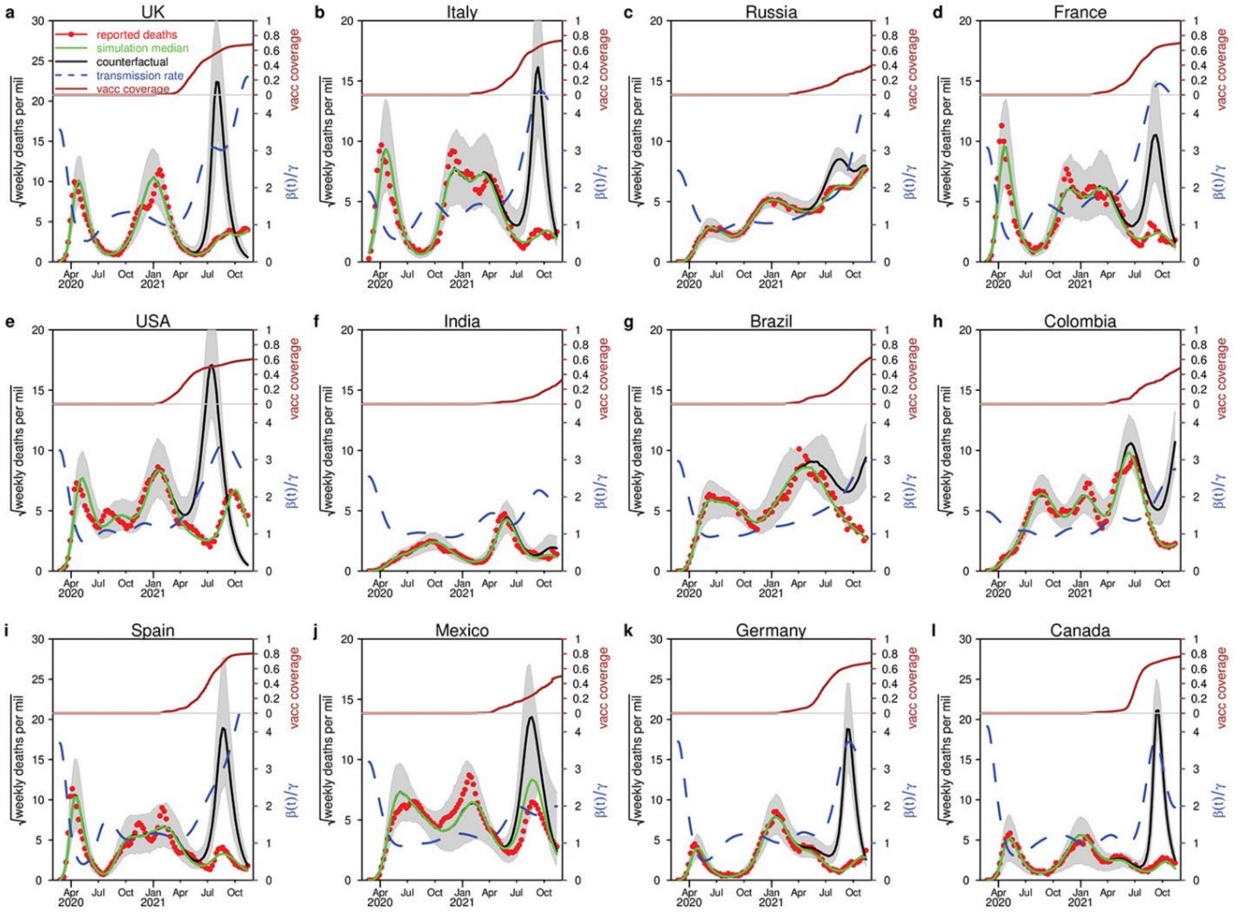

Appendix Figure 1. Modeled assessment of effectiveness of global COVID-19 vaccination campaign for 12 countries. We fit a state-space extended susceptible-exposed-infectious-recovered–type model (model 1) with a delayed class between infectious status to death and a death class in which η = 0.85, n_β = 10 to reported mortality data. Upper part of each panel shows the vaccination timing and real-time coverage (brown curve). Lower part of each panel shows reported COVID-19 deaths (red circles). Green curves indicated the median of 1,000 model simulations when vaccination was included in the model. Blue dashed curve shows the time varying transmission rate, $\beta(t)/\gamma$, as reconstructed by the model. Black curves show the counterfactual model simulations under the without-vaccination scenario when all other parameters are unchanged. Gray region indicates 95% CI of the simulations. The difference between green and black curves indicates the effects of vaccination in terms of saving lives (i.e., reduction in mortality) for these countries. Scales for the y-axes differ substantially to underscore patterns but do not permit direct comparisons. UK, United Kingdom; USA, United States.

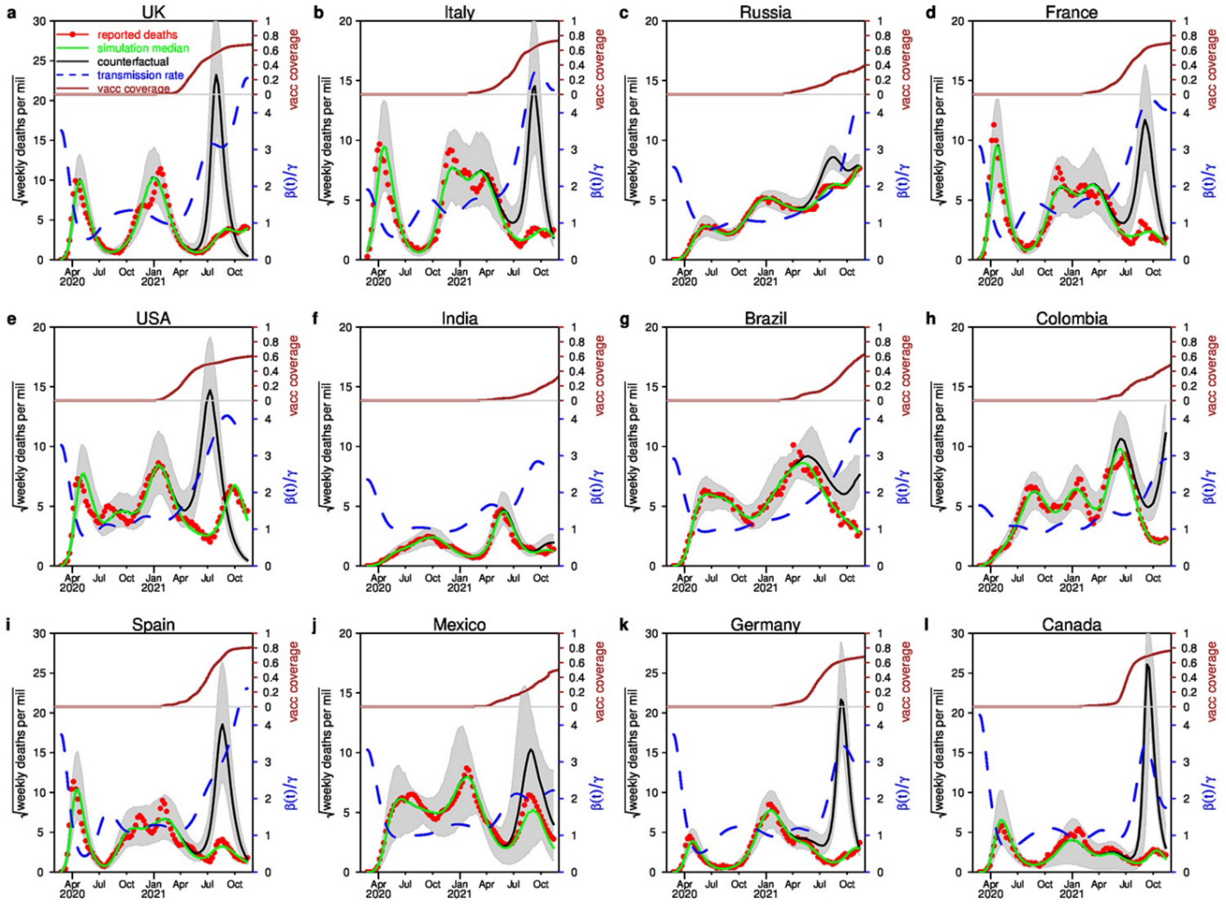

Appendix Figure 2. Modeled assessment of effectiveness of global COVID-19 vaccination campaign for 12 countries. We fit a state-space model to weekly reported mortality data. Results represent model 2 version 1 in which η = 0.85, ψ =0.6, and n_β = 10. Red circles are reported COVID-19 deaths. Brown curve shows the vaccination timing and real-time coverage. Green curve shows the median of 1,000 model simulations when vaccination is included in the model. Black curve shows the outcome under the first counterfactual scenario (i.e., $v(t)$ = 0); The gray region is the 95% confidence range of the simulations. The difference between green and black curves indicates the effects of vaccination in terms of deaths averted for these countries. Blue dashed curve shows the time varying transmission rate, $\beta(t)/\gamma$, as reconstructed by the model.

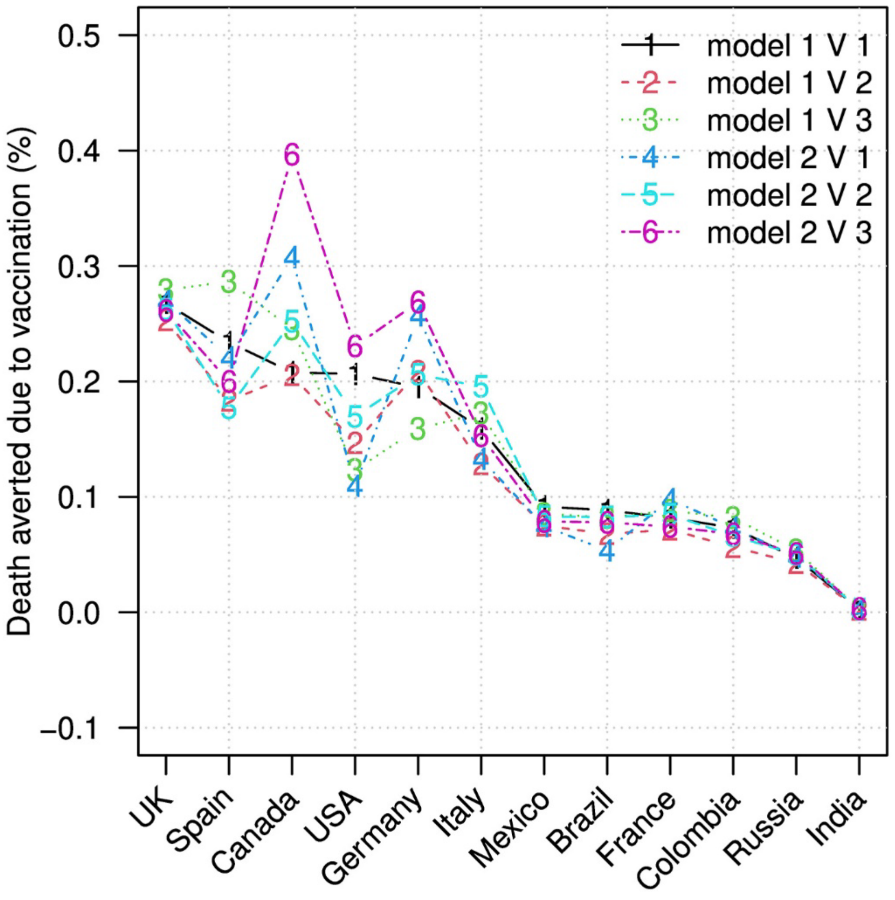

Appendix Figure 3. Death averted due to vaccination as a percentage of country's population as modeled assessment of effectiveness of global COVID-19 vaccination campaign for 12 countries. Against the first counterfactual scenario, we compare 6 model variations including the baseline model (model 1 version 1. UK, United Kingdom; USA, United States; V, version.
